# Supplementary material for: Migraine, Tension-Type Headache and Parkinson’s Disease: A Systematic Review and Meta-Analysis
Source: Medicina (Kaunas). 2022 Nov 20;58(11):1684. doi: 10.3390/medicina58111684 (PMC9697239; doi:10.3390/medicina58111684)
Supplement: Supplementary file 1 [file medicina-58-01684-s001.zip › Figure S1.pdf]

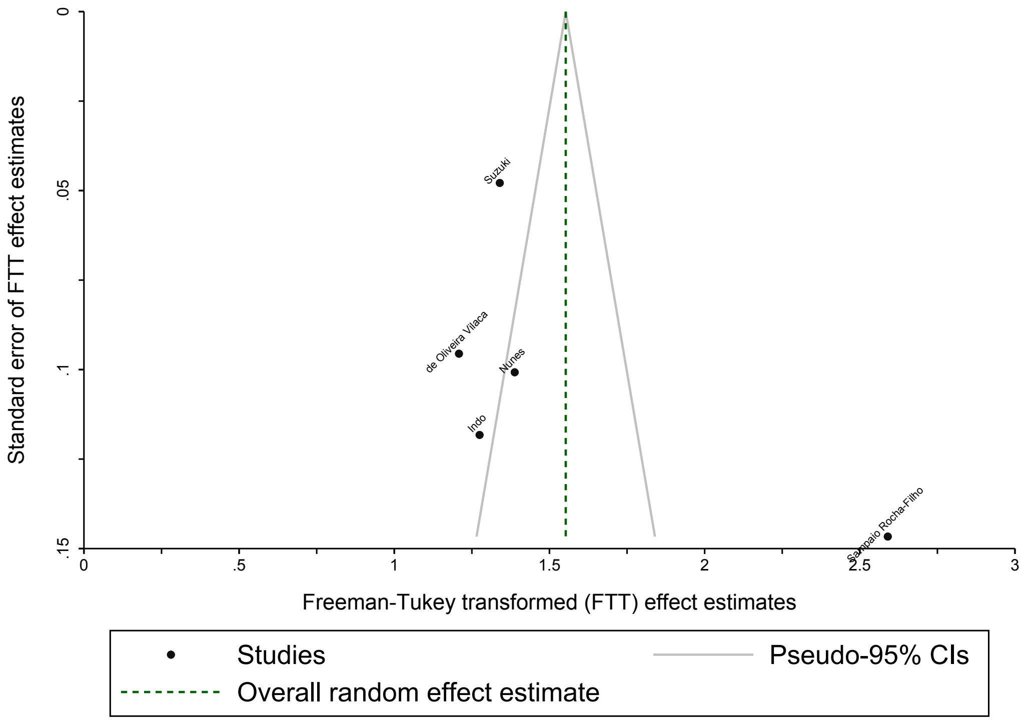

Standard error of FTT effect estimates
0
.05
.1
.15
0
.5
1
1.5
2
2.5
3
Freeman-Tukey transformed (FTT) effect estimates
Suzuki
de Oliveira Vilaca
Nunes
Indo
Sampaio Rocha-Filho
Studies
Pseudo-95% CIs
Overall random effect estimate

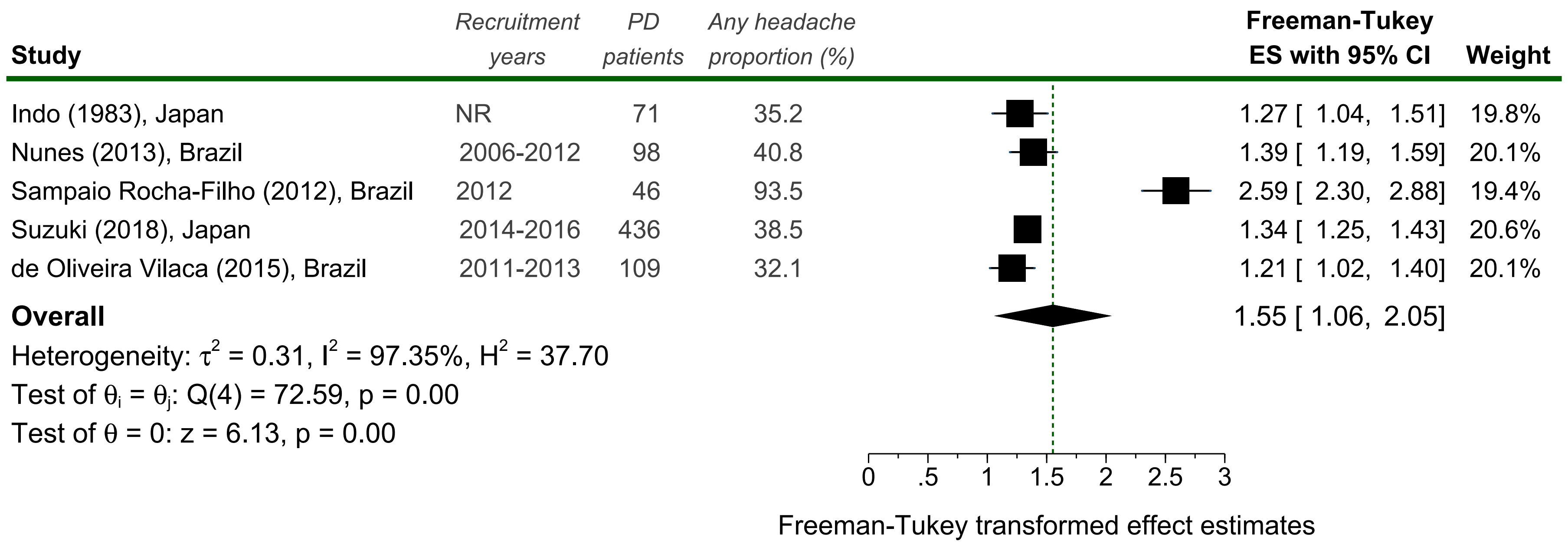

Study
Recruitment years
PD patients
Any headache proportion (%)
Freeman-Tukey ES with 95% CI
Weight
Indo (1983), Japan
NR
71
35.2
1.27 [1.04, 1.51]
19.8%
Nunes (2013), Brazil
2006-2012
98
40.8
1.39 [1.19, 1.59]
20.1%
Sampaio Rocha-Filho (2012), Brazil
2012
46
93.5
2.59 [2.30, 2.88]
19.4%
Suzuki (2018), Japan
2014-2016
436
38.5
1.34 [1.25, 1.43]
20.6%
de Oliveira Vilaca (2015), Brazil
2011-2013
109
32.1
1.21 [1.02, 1.40]
20.1%
Overall
1.55 [1.06, 2.05]
Heterogeneity: τ^2 = 0.31, I^2 = 97.35%, H^2 = 37.70
Test of $\theta_i = \theta_j$: Q(4) = 72.59, p = 0.00
Test of θ = 0: z = 6.13, p = 0.00
0
.5
1
1.5
2
2.5
3
Freeman-Tukey transformed effect estimates

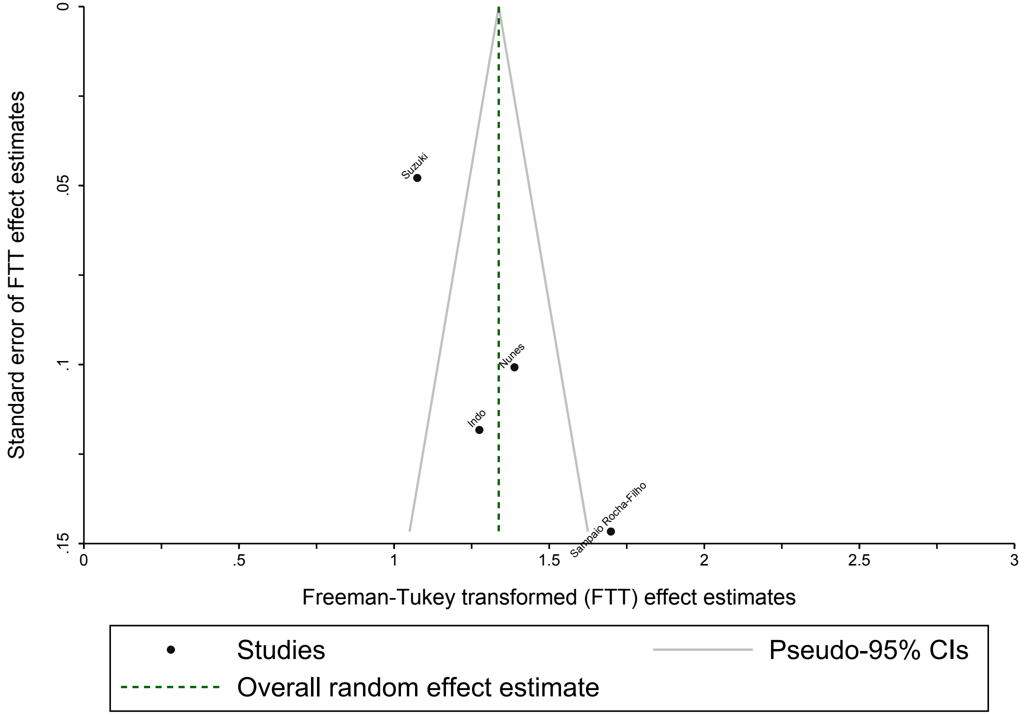

Standard error of FTT effect estimates
0
.05
.1
.15
Suzuki
Nunes
Indo
Sampaio Rocha-Filho
0
.5
1
1.5
2
2.5
3
Freeman-Tukey transformed (FTT) effect estimates
Studies
Pseudo-95% CIs
Overall random effect estimate

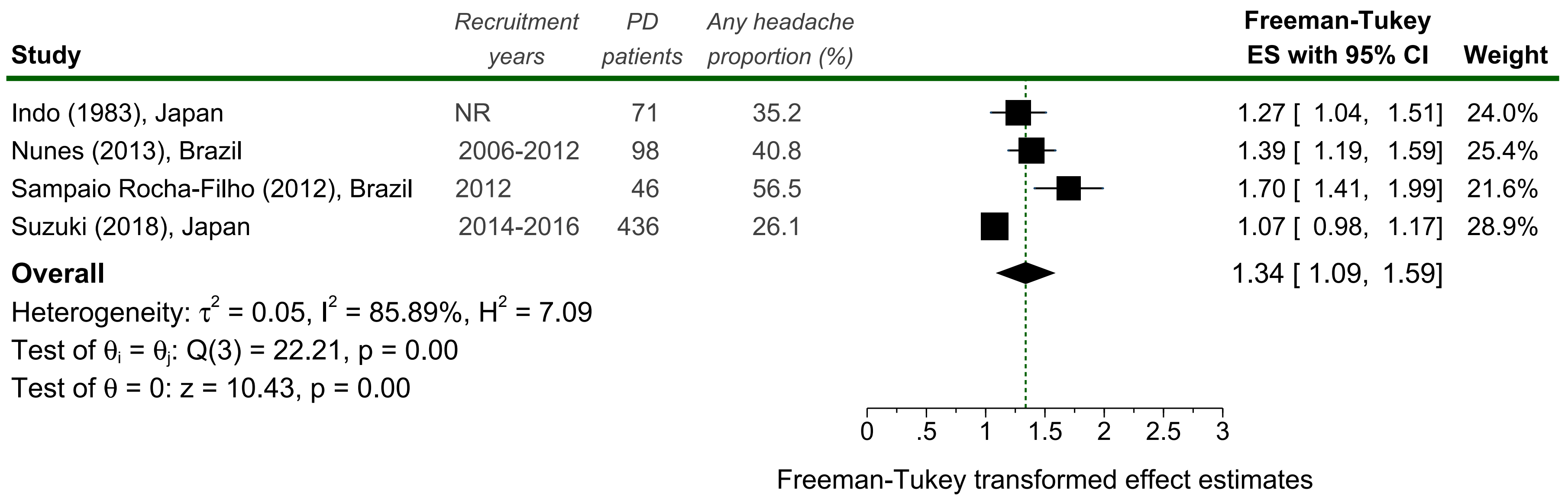
Study
Recruitment years
PD patients
Any headache proportion (%)
Freeman-Tukey ES with 95% CI
Weight
Indo (1983), Japan NR 71 35.2 1.27 [1.04, 1.51] 24.0%
Nunes (2013), Brazil 2006-2012 98 40.8 1.39 [1.19, 1.59] 25.4%
Sampaio Rocha-Filho (2012), Brazil 2012 46 56.5 1.70 [1.41, 1.99] 21.6%
Suzuki (2018), Japan 2014-2016 436 26.1 1.07 [0.98, 1.17] 28.9%
Overall 1.34 [1.09, 1.59]
Heterogeneity: τ^2 = 0.05, I^2 = 85.89%, H^2 = 7.09
Test of $\theta_i = \theta_j$: Q(3) = 22.21, p = 0.00
Test of θ = 0: z = 10.43, p = 0.00
0
.5
1
1.5
2
2.5
3
Freeman-Tukey transformed effect estimates

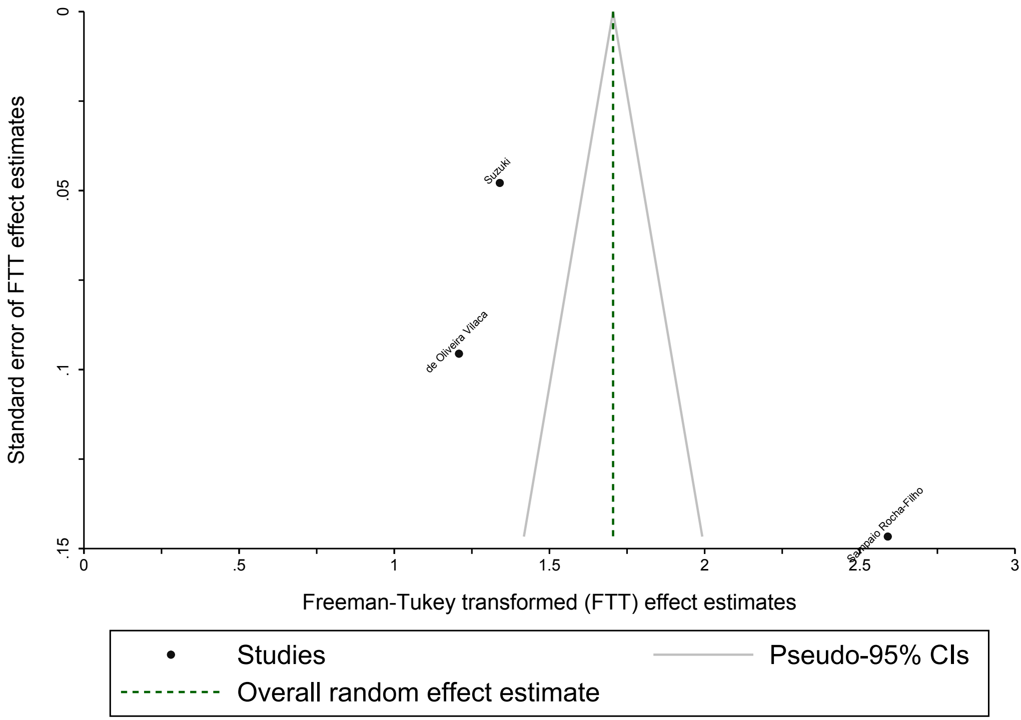

Standard error of FTT effect estimates
0
.05
.1
.15
Suzuki
de Oliveira Vilaca
Sampaio Rocha-Filho
0
.5
1
1.5
2
2.5
3
Freeman-Tukey transformed (FTT) effect estimates
Studies
Pseudo-95% CIs
Overall random effect estimate

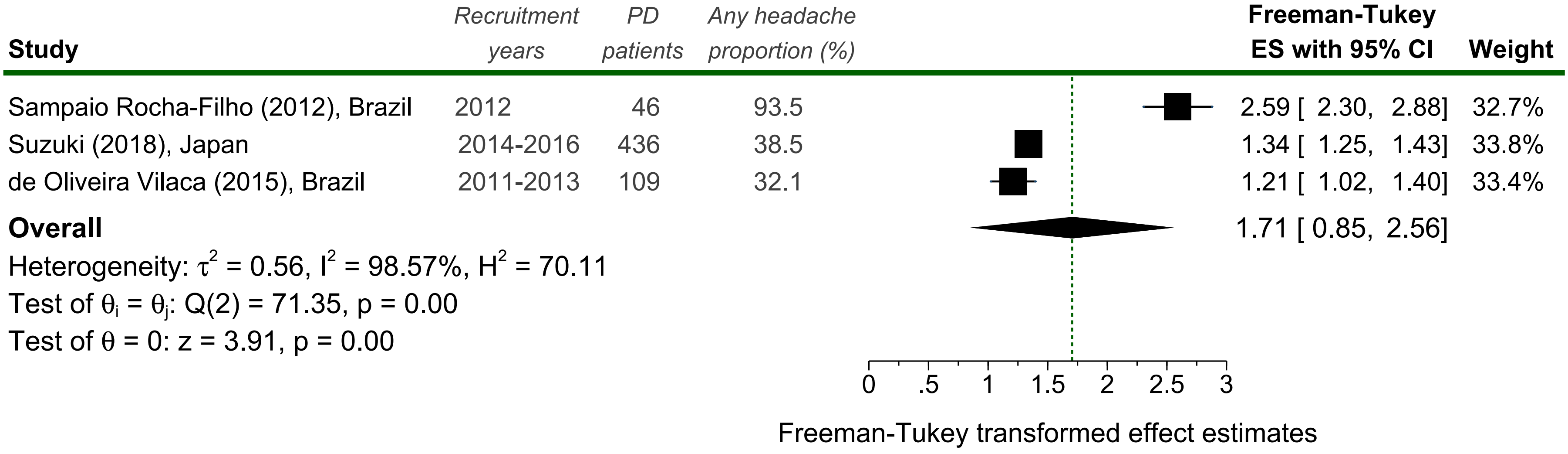
Study
Recruitment years
PD patients
Any headache proportion (%)
Freeman-Tukey ES with 95% CI
Weight
Sampaio Rocha-Filho (2012), Brazil
2012
46
93.5
2.59 [2.30, 2.88]
32.7%
Suzuki (2018), Japan
2014-2016
436
38.5
1.34 [1.25, 1.43]
33.8%
de Oliveira Vilaca (2015), Brazil
2011-2013
109
32.1
1.21 [1.02, 1.40]
33.4%
Overall
1.71 [0.85, 2.56]
Heterogeneity: τ^2 = 0.56, I^2 = 98.57%, H^2 = 70.11
Test of $\theta_i = \theta_j$: Q(2) = 71.35, p = 0.00
Test of θ = 0: z = 3.91, p = 0.00
0
.5
1
1.5
2
2.5
3
Freeman-Tukey transformed effect estimates

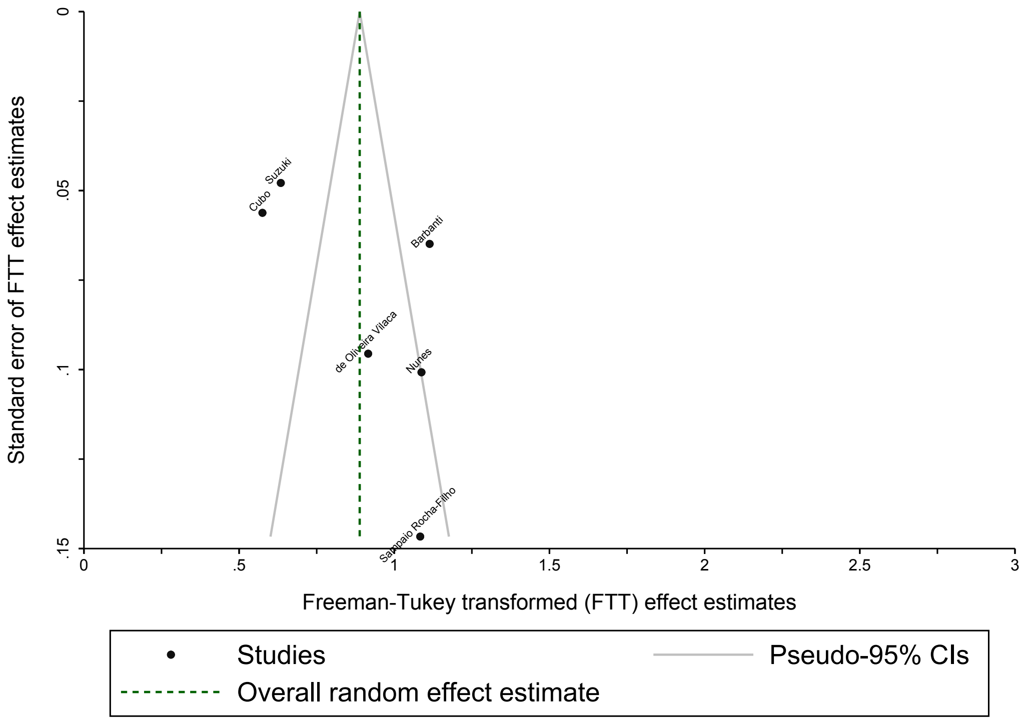

Standard error of FTT effect estimates
0
.05
.1
.15
Suzuki
Cubo
Barbanti
de Oliveira Vilaca
Nunes
Sampaio Rocha-Filho
0
.5
1
1.5
2
2.5
3
Freeman-Tukey transformed (FTT) effect estimates
Studies
Pseudo-95% CIs
Overall random effect estimate

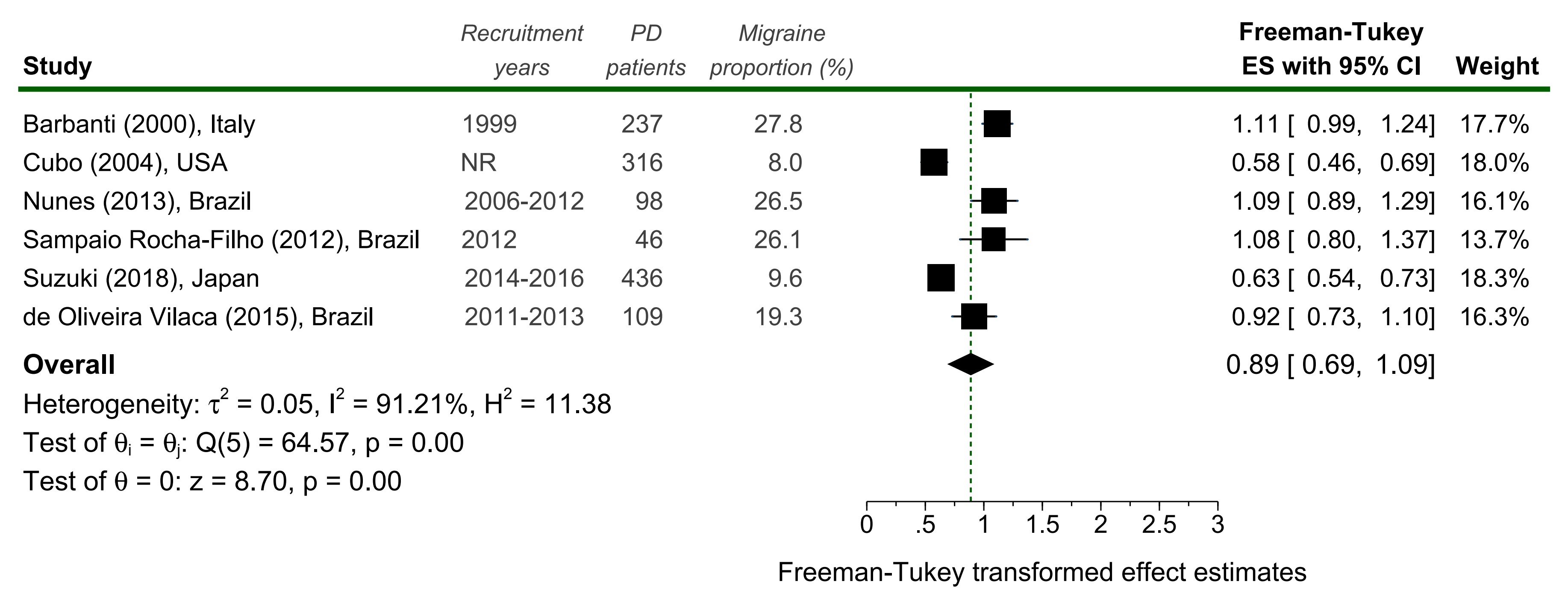
Study
Recruitment years
PD patients
Migraine proportion (%)
Freeman-Tukey ES with 95% CI
Weight
Barbanti (2000), Italy 1999 237 27.8 1.11 [0.99, 1.24] 17.7%
Cubo (2004), USA NR 316 8.0 0.58 [0.46, 0.69] 18.0%
Nunes (2013), Brazil 2006-2012 98 26.5 1.09 [0.89, 1.29] 16.1%
Sampaio Rocha-Filho (2012), Brazil 2012 46 26.1 1.08 [0.80, 1.37] 13.7%
Suzuki (2018), Japan 2014-2016 436 9.6 0.63 [0.54, 0.73] 18.3%
de Oliveira Vilaca (2015), Brazil 2011-2013 109 19.3 0.92 [0.73, 1.10] 16.3%
Overall 0.89 [0.69, 1.09]
Heterogeneity: τ^2 = 0.05, I^2 = 91.21%, H^2 = 11.38
Test of $\theta_i = \theta_j$: Q(5) = 64.57, p = 0.00
Test of θ = 0: z = 8.70, p = 0.00
0 .5 1 1.5 2 2.5 3
Freeman-Tukey transformed effect estimates

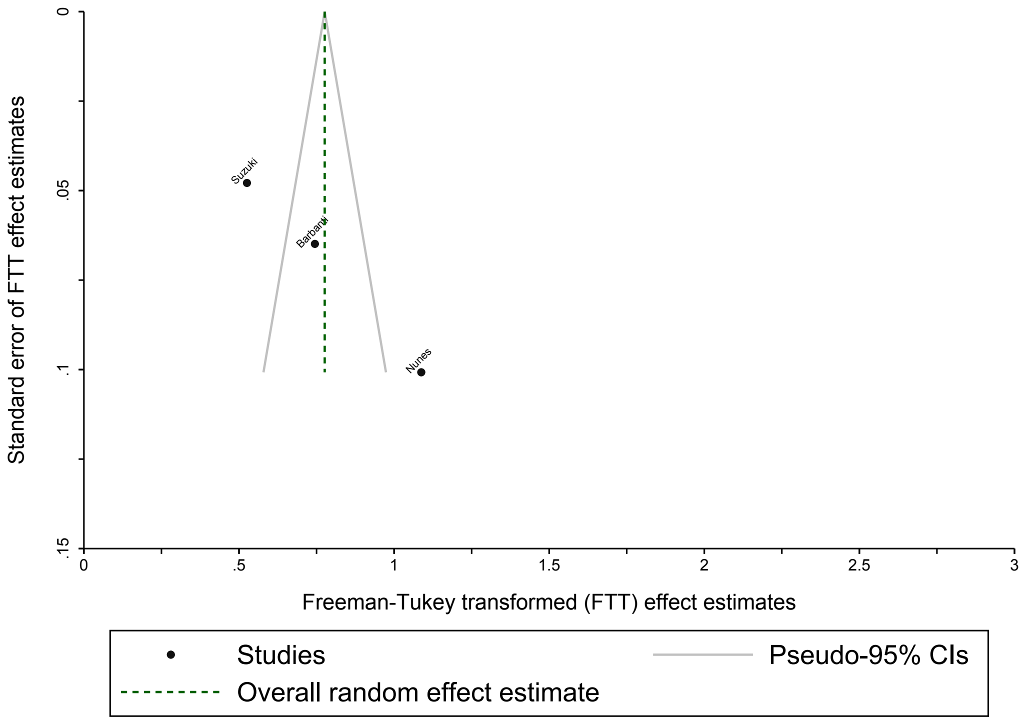

Standard error of FTT effect estimates
0
.05
.1
.15
Suzuki
Barbanti
Nunes
0
.5
1
1.5
2
2.5
3
Freeman-Tukey transformed (FTT) effect estimates
Studies
Pseudo-95% CIs
Overall random effect estimate

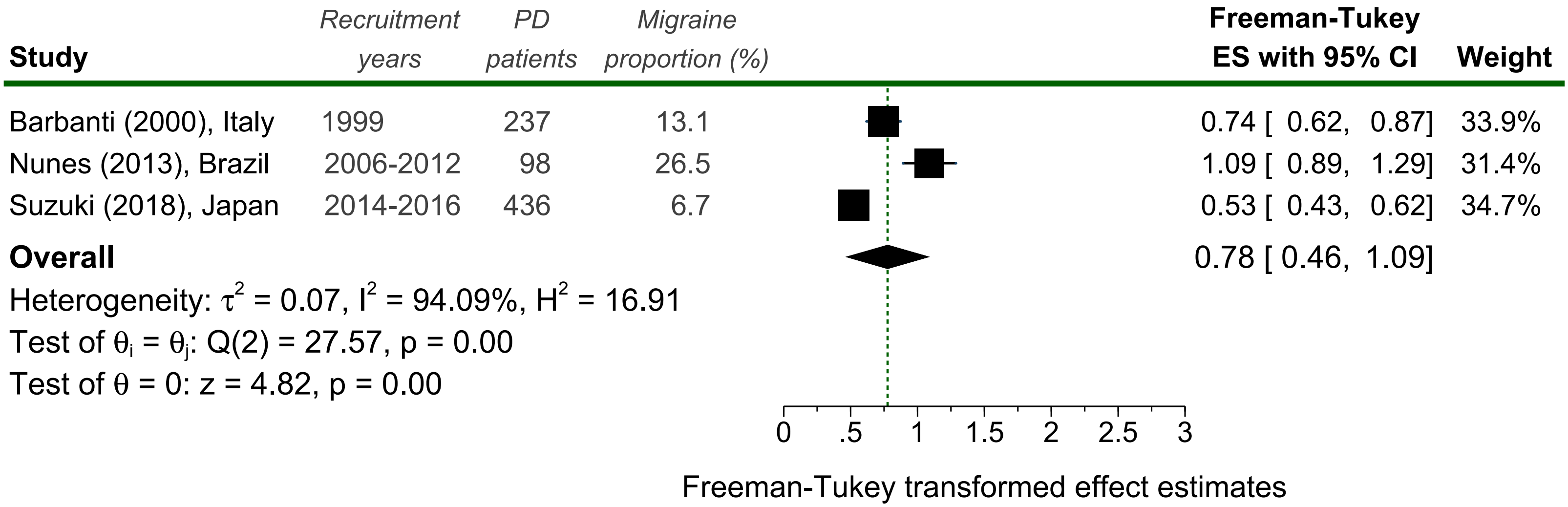
Study
Recruitment years
PD patients
Migraine proportion (%)
Freeman-Tukey ES with 95% CI
Weight
Barbanti (2000), Italy 1999 237 13.1 0.74 [0.62, 0.87] 33.9%
Nunes (2013), Brazil 2006-2012 98 26.5 1.09 [0.89, 1.29] 31.4%
Suzuki (2018), Japan 2014-2016 436 6.7 0.53 [0.43, 0.62] 34.7%
Overall 0.78 [0.46, 1.09]
Heterogeneity: τ^2 = 0.07, I^2 = 94.09%, H^2 = 16.91
Test of $\theta_i = \theta_j$: Q(2) = 27.57, p = 0.00
Test of θ = 0: z = 4.82, p = 0.00
0
.5
1
1.5
2
2.5
3
Freeman-Tukey transformed effect estimates

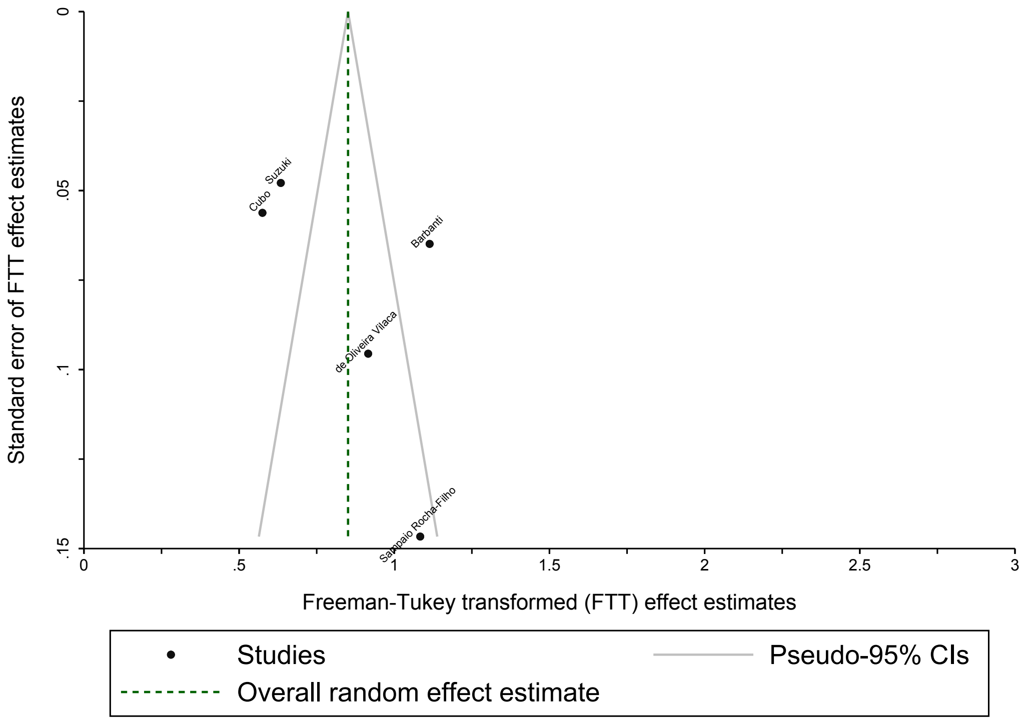

Standard error of FTT effect estimates
0
.05
.1
.15
Suzuki
Cubo
Barbanti
de Oliveira Vilaca
Sampaio Rocha-Filho
0
.5
1
1.5
2
2.5
3
Freeman-Tukey transformed (FTT) effect estimates
Studies
Pseudo-95% CIs
Overall random effect estimate

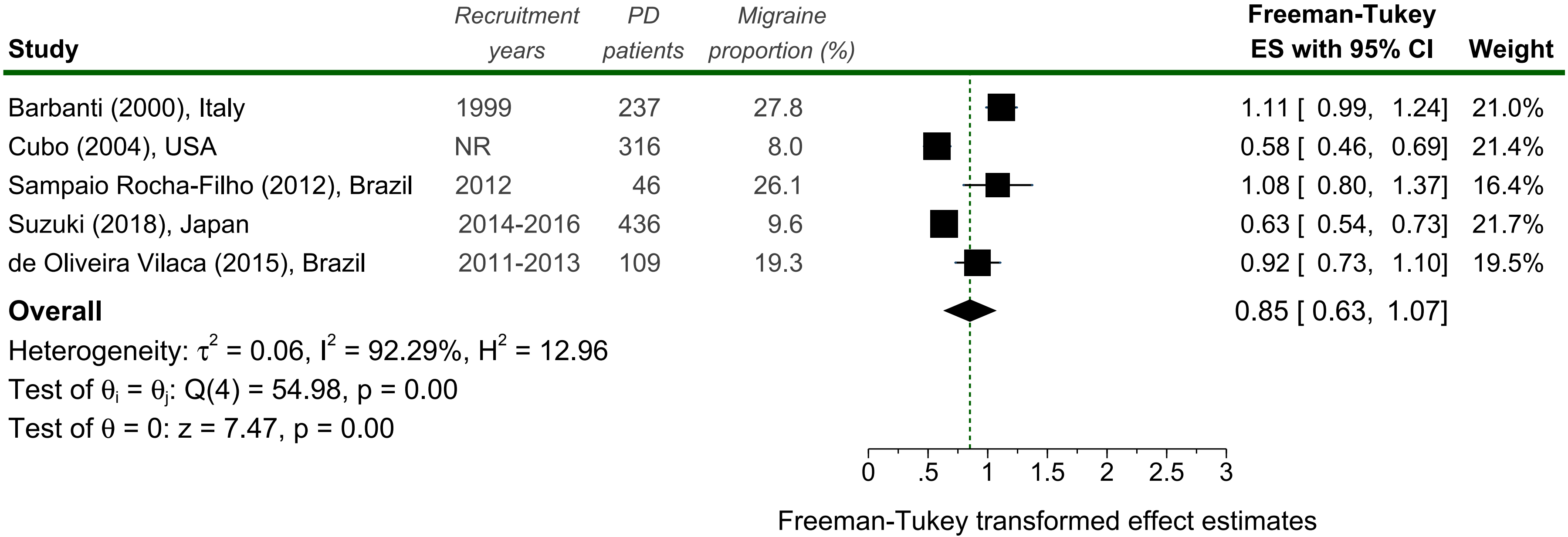
Study
Recruitment years
PD patients
Migraine proportion (%)
Freeman-Tukey ES with 95% CI
Weight
Barbanti (2000), Italy
1999
237
27.8
1.11 [0.99, 1.24]
21.0%
Cubo (2004), USA
NR
316
8.0
0.58 [0.46, 0.69]
21.4%
Sampaio Rocha-Filho (2012), Brazil
2012
46
26.1
1.08 [0.80, 1.37]
16.4%
Suzuki (2018), Japan
2014-2016
436
9.6
0.63 [0.54, 0.73]
21.7%
de Oliveira Vilaca (2015), Brazil
2011-2013
109
19.3
0.92 [0.73, 1.10]
19.5%
Overall
0.85 [0.63, 1.07]
Heterogeneity: τ^2 = 0.06, I^2 = 92.29%, H^2 = 12.96
Test of $\theta_i = \theta_j$: Q(4) = 54.98, p = 0.00
Test of θ = 0: z = 7.47, p = 0.00
0
.5
1
1.5
2
2.5
3
Freeman-Tukey transformed effect estimates

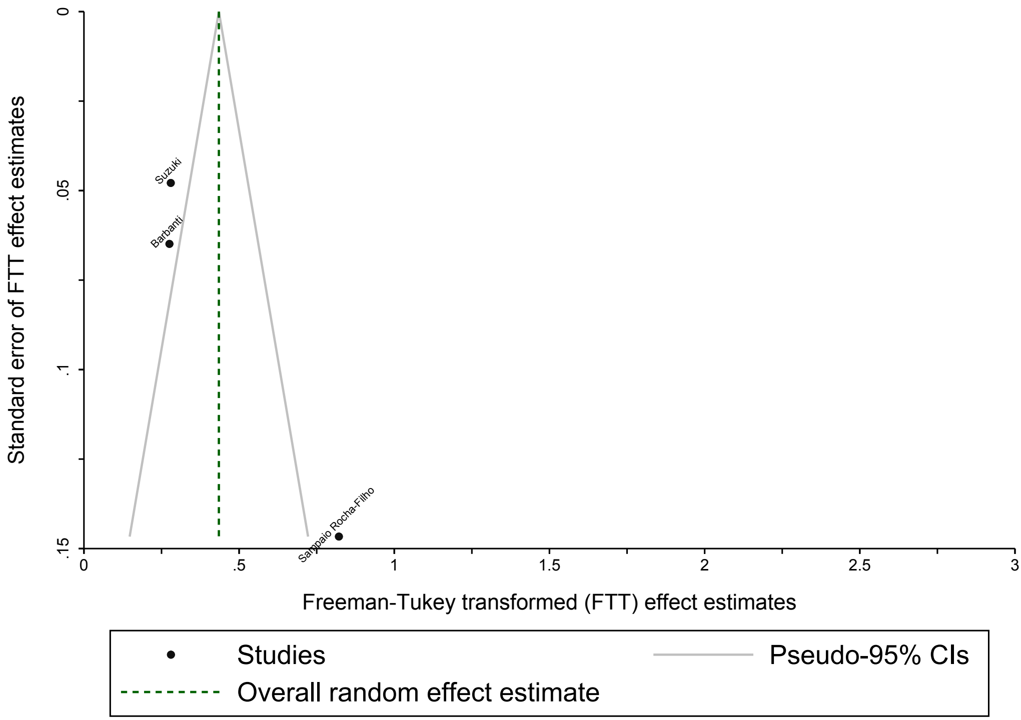

Standard error of FTT effect estimates
0
.05
.1
.15
Suzuki
Barbanti
Sampaio Rocha-Filho
0
.5
1
1.5
2
2.5
3
Freeman-Tukey transformed (FTT) effect estimates
Studies
Pseudo-95% CIs
Overall random effect estimate

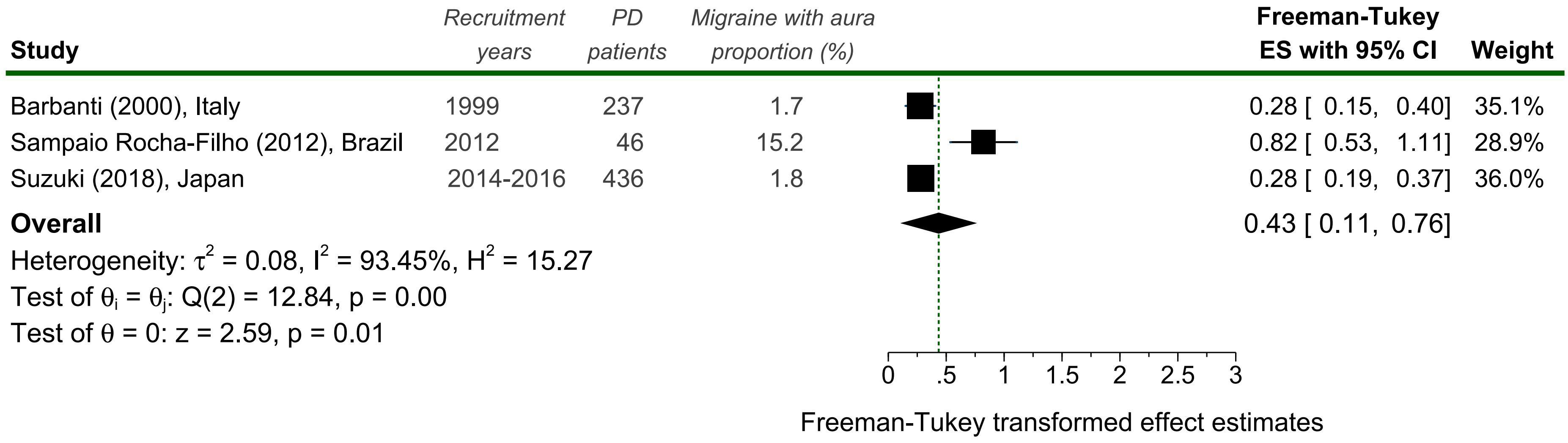

Study
Recruitment years
PD patients
Migraine with aura proportion (%)
Freeman-Tukey ES with 95% CI
Weight
Barbanti (2000), Italy
1999
237
1.7
0.28 [0.15, 0.40]
35.1%
Sampaio Rocha-Filho (2012), Brazil
2012
46
15.2
0.82 [0.53, 1.11]
28.9%
Suzuki (2018), Japan
2014-2016
436
1.8
0.28 [0.19, 0.37]
36.0%
Overall
0.43 [0.11, 0.76]
Heterogeneity: τ^2 = 0.08, I^2 = 93.45%, H^2 = 15.27
Test of $\theta_i = \theta_j$: Q(2) = 12.84, p = 0.00
Test of θ = 0: z = 2.59, p = 0.01
0
.5
1
1.5
2
2.5
3
Freeman-Tukey transformed effect estimates

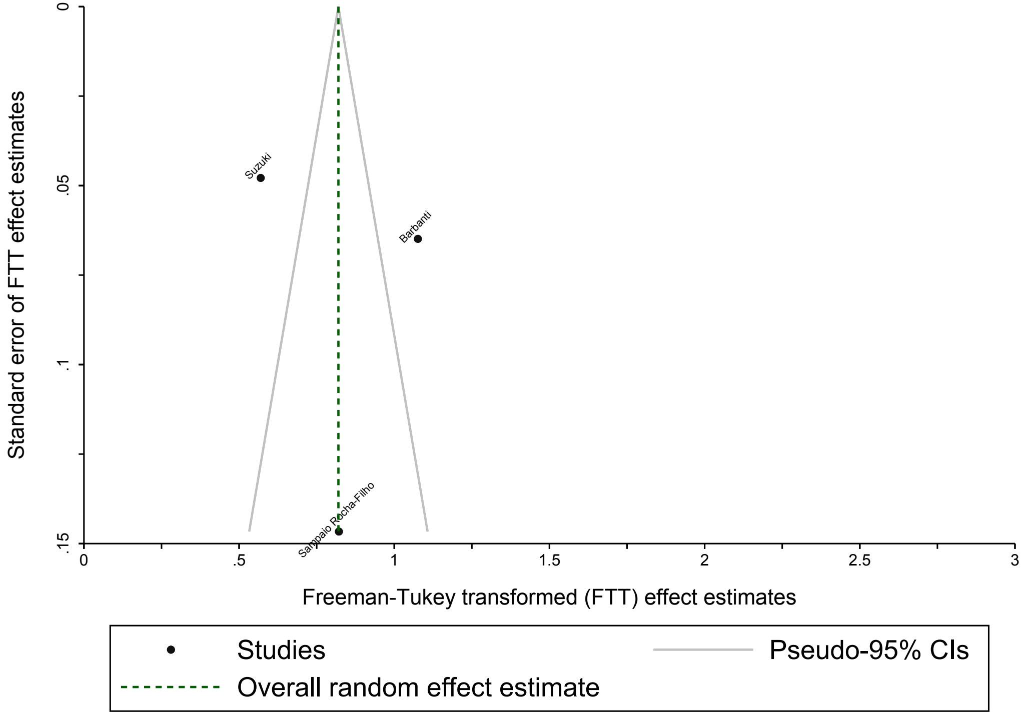

Standard error of FTT effect estimates
0
.05
.1
.15
Suzuki
Barbanti
Sampaio Rocha-Filho
0
.5
1
1.5
2
2.5
3
Freeman-Tukey transformed (FTT) effect estimates
Studies
Pseudo-95% CIs
Overall random effect estimate

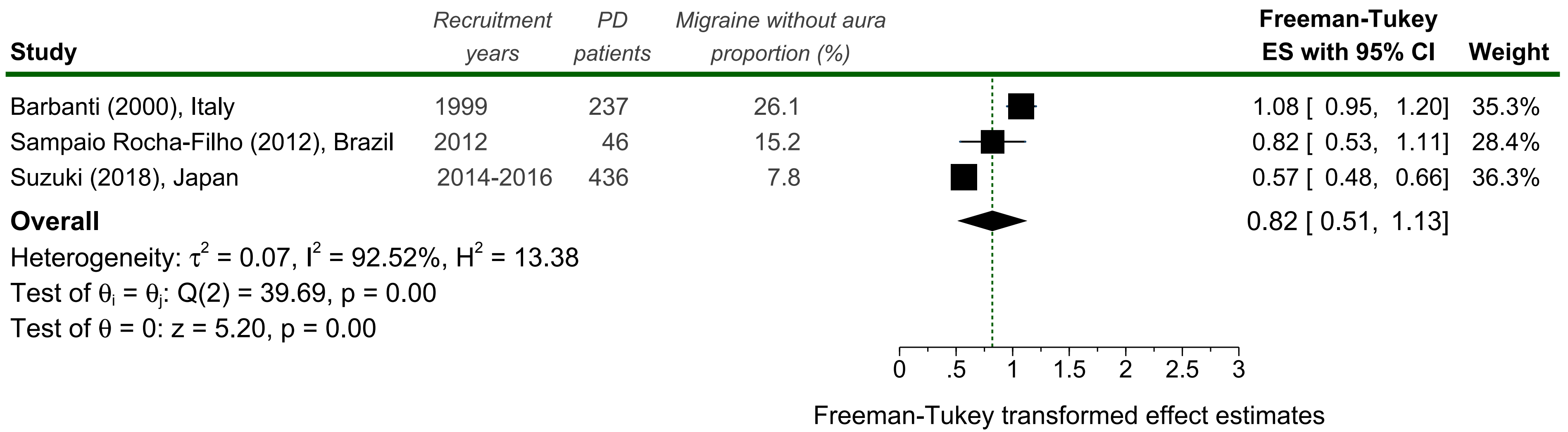
Study
Recruitment years
PD patients
Migraine without aura proportion (%)
Freeman-Tukey ES with 95% CI
Weight
Barbanti (2000), Italy 1999 237 26.1 1.08 [0.95, 1.20] 35.3%
Sampaio Rocha-Filho (2012), Brazil 2012 46 15.2 0.82 [0.53, 1.11] 28.4%
Suzuki (2018), Japan 2014-2016 436 7.8 0.57 [0.48, 0.66] 36.3%
Overall 0.82 [0.51, 1.13]
Heterogeneity: τ^2 = 0.07, I^2 = 92.52%, H^2 = 13.38
Test of $\theta_i = \theta_j$: Q(2) = 39.69, p = 0.00
Test of θ = 0: z = 5.20, p = 0.00
0
.5
1
1.5
2
2.5
3
Freeman-Tukey transformed effect estimates

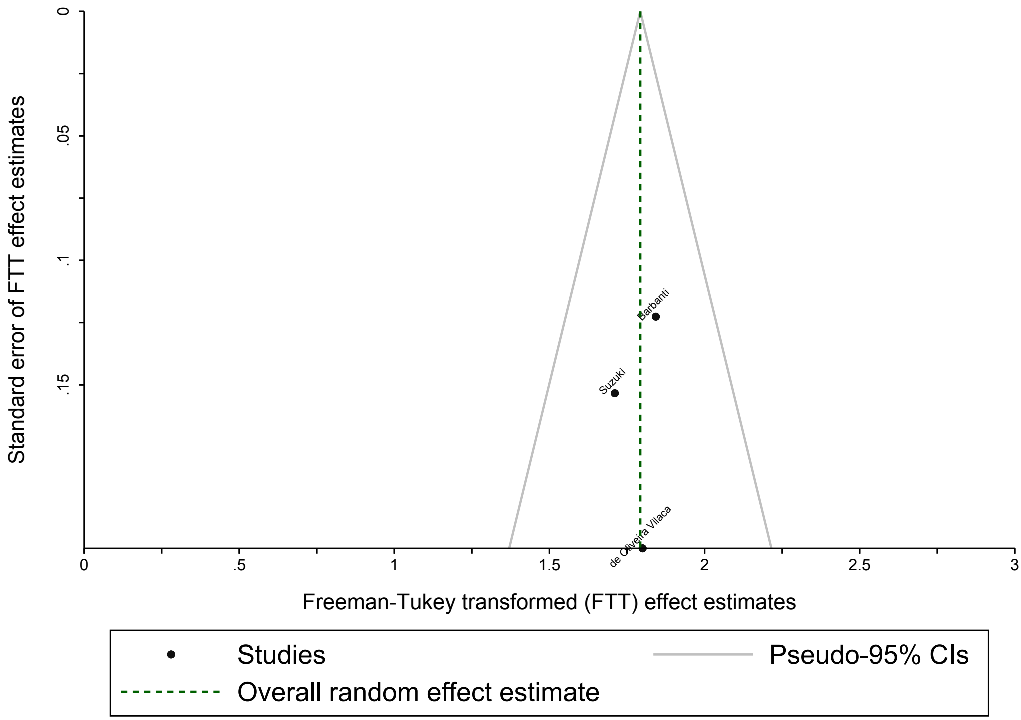

Standard error of FTT effect estimates
0
.05
.1
.15
0
.5
1
1.5
2
2.5
3
Barbanti
Suzuki
de Oliveira Vilaca
Freeman-Tukey transformed (FTT) effect estimates
Studies
Pseudo-95% CIs
Overall random effect estimate

Study	Recruitment years	PD patients	Headache improved proportion (%)	Freeman-Tukey ES with 95% CI	Weight
Barbanti (2000), Italy	1999	66	63.7	1.84 [1.60, 2.08]	51.0%
Suzuki (2018), Japan	2014-2016	42	57.1	1.71 [1.41, 2.01]	32.6%
de Oliveira Vilaca (2015), Brazil	2011-2013	21	62.0	1.80 [1.38, 2.22]	16.5%
**Overall**				1.79 [1.62, 1.96]	

Heterogeneity: $\tau^2 = 0.00$, $I^2 = 0.00\%$, $H^2 = 1.00$

Test of $\theta_i = \theta_j$: $Q(2) = 0.45$, $p = 0.80$

Test of $\theta = 0$: $z = 20.48$, $p = 0.00$

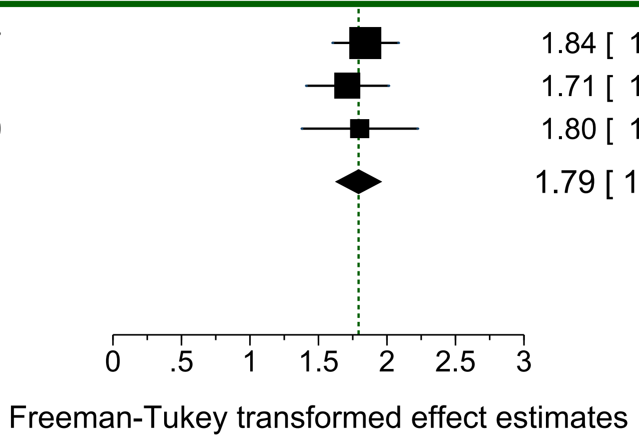

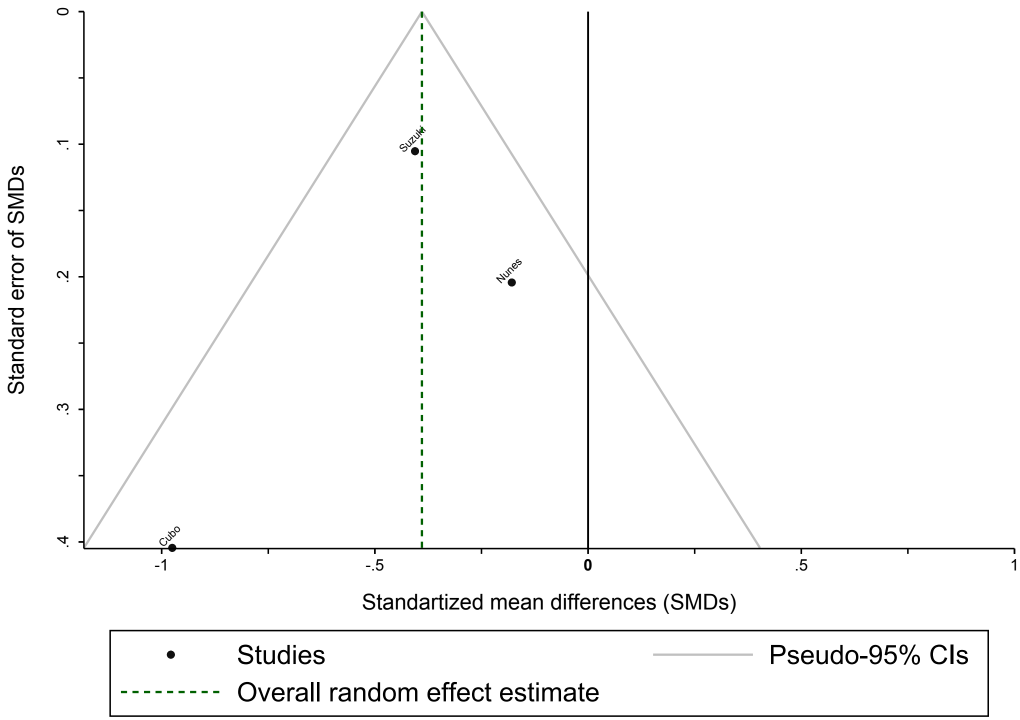
Standard error of SMDs
0
.1
.2
.3
.4
Suzuki
Nunes
Cubo
-1
-.5
0
.5
1
Standartized mean differences (SMDs)
Studies
Pseudo-95% CIs
Overall random effect estimate

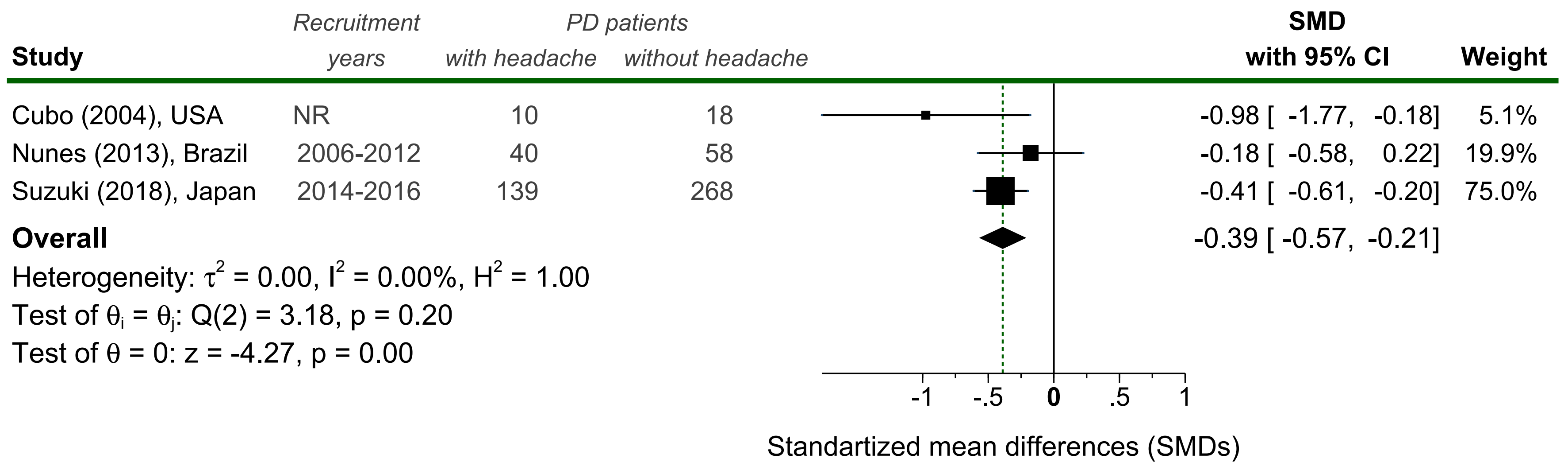

Recruitment years
PD patients
with headache
without headache
Study
SMD with 95% CI
Weight
Cubo (2004), USA NR 10 18 -0.98 [-1.77, -0.18] 5.1%
Nunes (2013), Brazil 2006-2012 40 58 -0.18 [-0.58, 0.22] 19.9%
Suzuki (2018), Japan 2014-2016 139 268 -0.41 [-0.61, -0.20] 75.0%
Overall -0.39 [-0.57, -0.21]
Heterogeneity: τ2 = 0.00, I2 = 0.00%, H2 = 1.00
Test of θi = θj: Q(2) = 3.18, p = 0.20
Test of θ = 0: z = -4.27, p = 0.00
-1
-.5
0
.5
1
Standartized mean differences (SMDs)

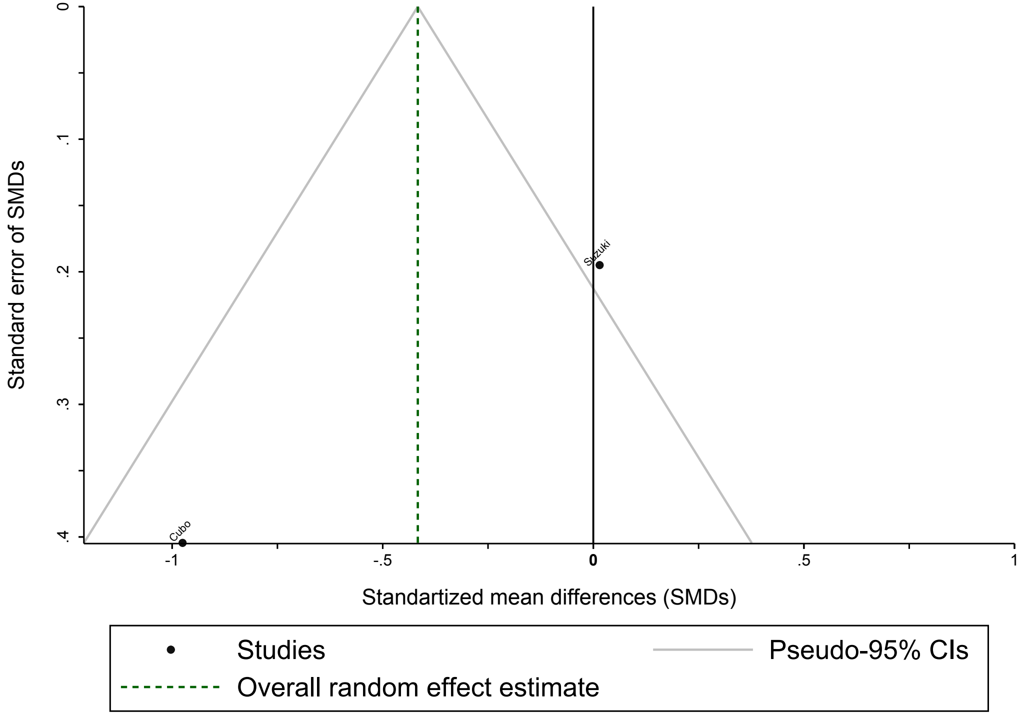

Standard error of SMDs
0
.1
.2
.3
.4
Suzuki
Cubo
-1
-.5
0
.5
1
Standartized mean differences (SMDs)
Studies
Pseudo-95% CIs
Overall random effect estimate
