# Supplementary material for: Migraine, Tension-Type Headache and Parkinson’s Disease: A Systematic Review and Meta-Analysis
Source: Medicina (Kaunas). 2022 Nov 20;58(11):1684. doi: 10.3390/medicina58111684 (PMC9697239; doi:10.3390/medicina58111684)
Supplement: Supplementary file 1 [file medicina-58-01684-s001.zip › Table S1.pdf]

Table S1 Quality evaluation of case-control studies according to the Newcastle-Ottawa Scale (NOS) and modified NOS (for cross-sectional studies)

Cross-sectionalStudies	Selection	Comparability	Exposure	NOS score
**Indo, 1983**	*		**	3/9
**Barbanti,2000**	***	*	**	6/9
**Cubo,2004**	***	*	**	6/9
**de OliveiraVilaça, 2015**	***	*	**	6/9
**SampaioRocha-Filho,2020**	***	*	**	6/9
**Nunes,2013**	***	*	**	6/9
**Suzuki, 2018**	***	*	**	6/9
**Case-Control Studies**				
**Meco, 1988**	*	**	**	5/9
**Heilbron,2019**	***	*	**	6/9
**de OliveiraVilaça, 2015#**	***	*	**	6/9
**Nunes,2013#**	***	*	**	6/9
**Suzuki, 2018#**	***	*	**	6/9
**Prospective Cohort Studies**				
**Scher, 2014**	***	**	**	7/9
**Wang,2016**	***	**	**	7/9
**Yang, 2017**	***	**	**	7/9

Selection: a: Representativeness of the exposed cohort; b: Selection of the non-exposed cohort; c: Ascertainment of exposure; D: Demonstration that outcome of interest was not present at start of study Comparability: e:Comparability of Cohorts on Basis of Design or Analysis Time to Follow-Up Exposure: f: Assessment of outcome; g: Follow-up long enough for outcomes to occur; h: Adequacy of follow-up of cohorts .The number of * corresponds to the number of items assessed positively in each category #these studies are included in more than one study design because different outcomes correspond to different designs (prevalence-cross sectional and association estimates-case control).
